# Venous thromboembolism in viral diseases: A comprehensive literature review

**DOI:** 10.1002/hsr2.1085

**Published:** 2023-02-05

**Authors:** Nasibeh Zerangian, Gisou Erabi, Mohadeseh Poudineh, Kosar Monajjem, Maryam Diyanati, Maryam Khanlari, Amirmohammad Khalaji, Diba Allafi, Arezoo Faridzadeh, Arian Amali, Nilufar Alizadeh, Yasaman Salimi, Sajjad Ghane Ezabadi, Amir Abdi, Zahra Hasanabadi, Mahdie ShojaeiBaghini, Niloofar Deravi

**Affiliations:** ^1^ Health Education and Health Promotion, Department of Health Education and Health Promotion, School of Health Mashhad University of Medical Sciences Mashhad Iran; ^2^ Student Research Committee Urmia University of Medical Sciences Urmia Iran; ^3^ School of Medicine Zanjan University of Medical Sciences Zanjan Iran; ^4^ Student Research Committee Tabriz University of Medical Sciences Tabriz Iran; ^5^ Student Research Committee Rafsanjan University of Medical Sciences Rafsanjan Iran; ^6^ School of Medicine Tehran University of Medical Sciences Tehran Iran; ^7^ Department of Immunology and Allergy, School of Medicine Mashhad University of Medical Sciences Mashhad Iran; ^8^ Immunology Research Center Mashhad University of Medical Sciences Mashhad Iran; ^9^ Student Research Committee, Paramedical Department Islamic Azad University, Mashhad Branch Mashhad Iran; ^10^ Doctor of Medicine (MD), School of Medicine Iran University of Medical Sciences Tehran Iran; ^11^ Student Research Committee Kermanshah University of Medical Sciences Kermanshah Iran; ^12^ Student's Scientific Research Center, School of Medicine Tehran University of Medical Sciences Tehran Iran; ^13^ Student Research Committee, School of Medicine, Tehran Medical Sciences Islamic Azad University Tehran Iran; ^14^ Doctor of Medicine (MD), School of Medicine Qazvin University of Medical Science Qazvin Iran; ^15^ Medical Informatics Research Center, Institute for Futures Studies in Health Kerman University of Medical Sciences Kerman Iran; ^16^ Student Research Committee, School of Medicine Shahid Beheshti University of Medical Sciences Tehran Iran

**Keywords:** COVID‐19, venous thromboembolism, viral diseases, VTE

## Abstract

Venous thromboembolism (VTE) is known to be a common respiratory and/or cardiovascular complication in hospitalized patients with viral infections. Numerous studies have proven human immunodeficiency virus infection to be a prothrombotic condition. An elevated VTE risk has been observed in critically ill H1N1 influenza patients. VTE risk is remarkably higher in patients infected with the Hepatitis C virus in contrast to uninfected subjects. The elevation of D‐dimer levels supported the association between Chikungunya and the Zika virus and the rise of clinical VTE risk. Varicella‐zoster virus is a risk factor for both cellulitis and the consequent invasive bacterial disease which may take part in thrombotic initiation. Eventually, hospitalized patients infected with the coronavirus disease of 2019 (COVID‐19), the cause of the ongoing worldwide pandemic, could mainly suffer from an anomalous risk of coagulation activation with enhanced venous thrombosis events and poor quality clinical course. Although the risk of VTE in nonhospitalized COVID‐19 patients is not known yet, there are a large number of guidelines and studies on thromboprophylaxis administration for COVID‐19 cases. This study aims to take a detailed look at the effect of viral diseases on VTE, the epidemiology of VTE in viral diseases, and the diagnosis and treatment of VTE.

## 1 INTRODUCTION

Venous thromboembolism (VTE), consisting of deep vein thrombosis (DVT) and pulmonary embolism (PE), is a common, significant disease whose frequency is about 1 per 1000 person‐years in the overall population.^1^ An approximate cost of 4.9–19.8 billion dollars annually is spent on that in America.^2^ Since its frequency and risk factors are increasing, prevention and treatment of VTE are of special importance. Additionally, VTE is a preventable disease and, it is crucial to identify high‐risk individuals as they often remain unrecognized, so they may benefit from the primary prophylaxis.^1^, ^3^

One of the known risk factors is bacterial and viral infections due to the systemic inflammation that they cause.^4^ Human immunodeficiency virus (HIV) leads to an increase of about 2‐ to 10‐fold in VTE risk in HIV‐positive patients.^5^ Although there is little documentation backing the association between Zika and Chikungunya virus and VTE, we should consider the possibility of that in more severe cases as inflammatory processes and immobilization in these patients are overall trigger factors for the progress of VTE.^6^, ^7^

Some supplementary studies have reported DVT and cerebral venous thrombosis associated with Herpes Simplex Virus (HSV).^8^, ^9^ Hepatitis C virus (HCV) infection accompanies VTE with a prevalence of 0.8%.^10^ The most frequent site of vein thrombosis is the portal vein in cirrhotic patients.^11^, ^12^ VTE is also one of the hematological complications of infectious mononucleosis, although uncommon in immunocompetent patients.^13^ In children with acute Varicella zoster virus (VZV) infection, PE and DVT have been frequently reported.^14^ It can also cause stroke in children who suffer from chickenpox.^15^ It has been recommended to be watchful for PE in patients with acute respiratory symptoms during the epidemy of influenza.^16^ In December 2019, severe acute respiratory syndrome coronavirus 2 (SARS CoV‐2) appeared as a novel pathogen causing viral pneumonia. Billions of people have been affected worldwide, and it has been responsible for more than 350,000 deaths. Thromboembolic complications, which are manifested by DVT, PE, cerebrovascular accident (CVA), limb and mesenteric arterial ischemia, and myocardial infarction (MI) are some of the significant reasons for death.^17^, ^18^ It can also happen in asymptomatic patients.^19^

Different theories have described the interplay between viral infections, the coagulation pathway, and the hemostasis system. Most of these theories are based on endothelial inflammation and injury caused by uncontrolled viral replication.^13^, ^17^, ^20^, ^21^ For instance, pro‐inflammatory cytokines produced by Epstein–Barr virus (EBV) and coronavirus disease of 2019 (COVID‐19),^13^, ^22^ the endothelial infection caused by Cytomegalovirus (CMV) and EBV, elevated levels of Von‐Willebrand factor because of COVID‐19 and CMV,^17^, ^21^ diminished activity of the protein C pathway accrues in pneumonia caused by the influenza virus^23^ can lead to thrombosis formation. Some items have been suggested as VTE risk factors in each viral infection. Older age and male gender in the HCV infection,^10^ higher viral load, low CD4^+^ count, and presence of opportunistic infection such as CMV, malignancy, and thrombophilias in the HIV infection are known mechanisms behind the crosstalk existing between the inflammation and hemostasis system.^3^, ^24^, ^25^ However, more research should be considered to better clarify the associations between viral infections and the hemostasis system.

In‐hospital immobilization and current smoking are risk factors for VTE in influenza infection.^16^ Moreover, oral contraceptives, cancer, obesity, immobilization, diabetes, and lupus anticoagulant (LAC) was related to CMV‐associated DVT. There are also some genetic factors such as Factor V G1691A and factor VIII mutation.^4^, ^26^, ^27^ Age has a direct association with the pervasiveness of VTE, PE, and DVT in COVID‐19, while body mass index (BMI) affects only PE prevalence. Male sex does not count as a risk factor based on a meta‐analysis.^28^ Other risk factors in COVID‐19‐associated thromboembolism are immobilization, hypertension, intensive care unit (ICU) admission, central vein catheters, tissue factors, and cytokines.^22^

VTE diagnosis primarily rests on two bases, including clinical suspicion and then paraclinical confirmation,^5^, ^15^ which can be accomplished by Doppler ultrasound as the initial imaging exam.^24^, ^29^ Molecular genetic testing could be considered if one is suspicious of underlying coagulopathies.^24^, ^29^ D‐dimer is also a reliable parameter; however, it is not specific for VTE. In COVID‐19 patients, it has been accounted a biomarker indicating coagulopathy and prognosis of the disease.^20^, ^22^

Unfractionated heparin along with low‐molecular‐weight heparin (LMWH) is the most common therapies used for treatment and prophylaxis in viral‐associated VTE such as Zika virus, SARS‐CoV‐2, and other viral infections causing VTE. If there is any contraindication, mechanical prophylaxis should be considered.^19^ In HIV‐infected patients, guidelines recommend anticoagulant therapy for those with persisting risk factors.^5^, ^30^ Patients with CMV‐associated VTE need to receive anticoagulant therapy even if only small vessels are involved. Although no guideline has already clarified the management, clinicians usually administrate LMWH and warfarin.^4^, ^31^ Empirical anticoagulation can protect significantly from VTE in H1N1‐positive patients; particularly, prophylaxis has been recommended in high‐risk patients.^32^ Prescribing prophylaxis in COVID‐19 patients depends on different factors, and clinicians would better individualize each case. However, a direct‐acting oral anticoagulant (DOAC) is usually administered in a prophylactic dose such as Rivaroxaban, Apixaban, or Betrixaban.^33^

In this review article, we seek to overview and outline the epidemiology, pathophysiology, diagnostic methods, prophylaxis, and treatment approaches to VTE caused by viral infections to easily compare these issues in different viruses. For this purpose, we reviewed case reports, case controls, review articles, systematic reviews, and cohort studies.

## 2 LITERATURE SEARCH

**Figure 1:**
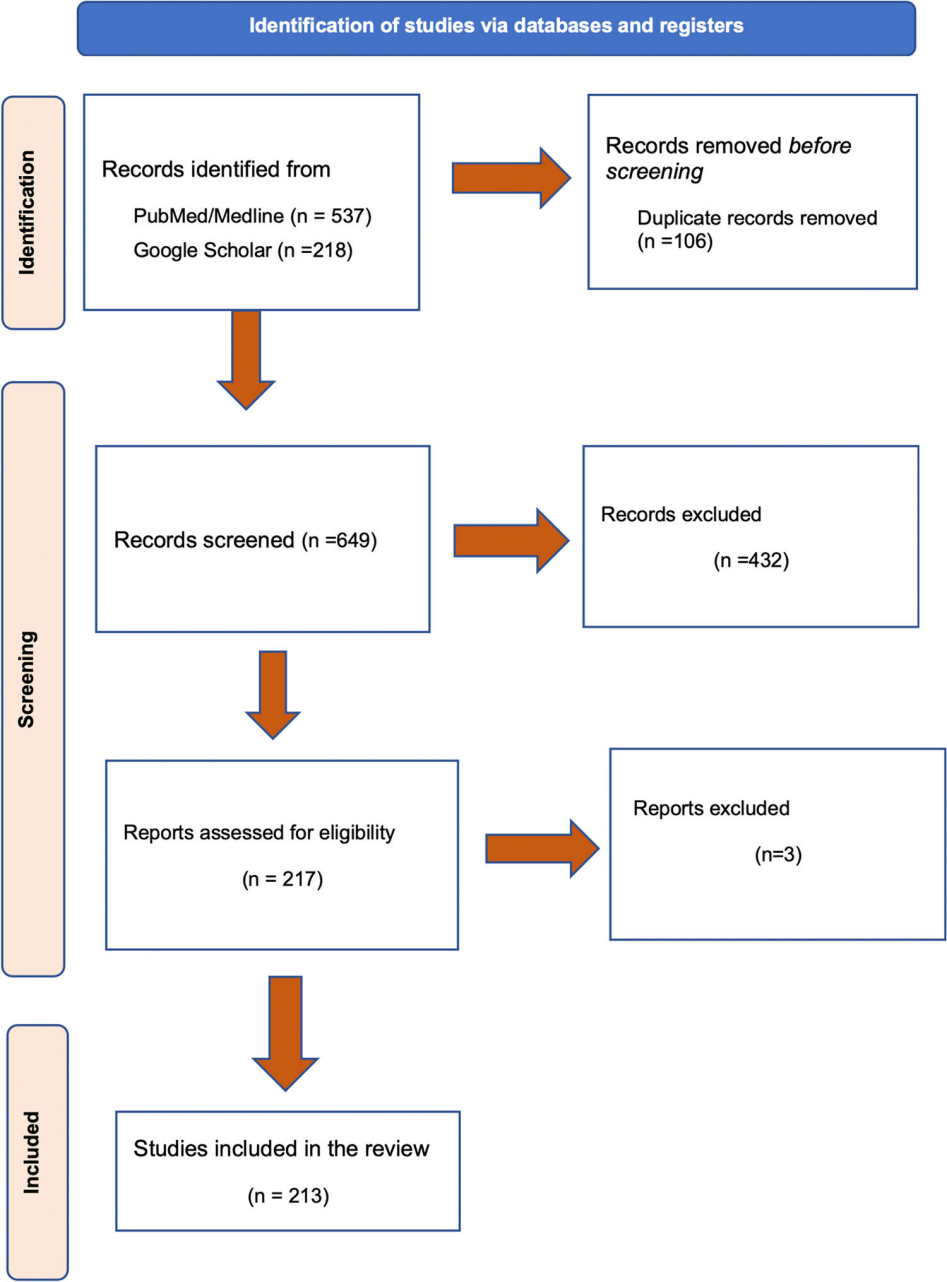
The selection process of studies included in this review.

This study was conducted by searching Google Scholar and PubMed database from inception up to December 2022. Summaries were presented and discussed VTE. The search keywords comprised VTE, Thromboembolisms, Thrombosis, DVT, PE, HIV, Herpes simplex virus, Hepatitis C virus, Zika virus, Chikungunya virus, Epstein Barr virus, Varicella zoster, Influenza (H1N1), Cytomegalovirus, Q fever virus, SARS‐VoV2, and COVID‐19. Table 1 summarized the keywords searched in Google Scholar and PubMed online databases. Inclusion criteria were in vitro, in vivo, or clinical studies including case series, observational studies, and randomized controlled trials (RCTs) that evaluated the roles of viral infections in VTE. We excluded non‐English studies and studies that were not related to the effects of viral infections in VTE. Figure 1 illustrated the selection process of studies included in this review. The references list of included articles was also searched manually for possible relevant studies.

**Table 1:** Search strategies for PubMed and Google scholar.

Search engine	Search strategy	Additional filters
PubMed/MEDLINE	(Venous Thromboembolism [Title/Abstract] OR Thromboembolisms [Title/Abstract] OR Thrombosis [Title/Abstract] OR deep vein thrombosis [Title/Abstract] OR pulmonary embolism [Title/Abstract]) AND (HIV [Title/Abstract] OR Herpes simplex virus [Title/Abstract] OR Hepatitis C virus [Title/Abstract] OR Zika virus [Title/Abstract] OR Chikungunya virus [Title/Abstract] OR Epstein Barr virus [Title/Abstract] OR Varicella zoster [Title/Abstract] OR Influenza (H1N1 [Title/Abstract]) OR Cytomegalovirus [Title/Abstract] OR Q fever virus [Title/Abstract] OR Sars‐cov2[Title/Abstract] OR COVID‐19 virus [Title/Abstract])	English, December 1st 2022
Google scholar	VenousThromboembolism or Thromboembolisms or Thrombosis or deepveinthrombosis or pulmonaryembolism and HIV or Herpessimplexvirus or HepatitisCvirus or Zikavirus or Chikungunyavirus or EpsteinBarrvirus or Varicellazoster or Influenza(H1N1) or Cytomegalovirus or Qfevervirus or Sars‐cov2 or COVID‐19virus	English, December 1st 2022

## 3 HIV AND VTE

**Figure 2:**
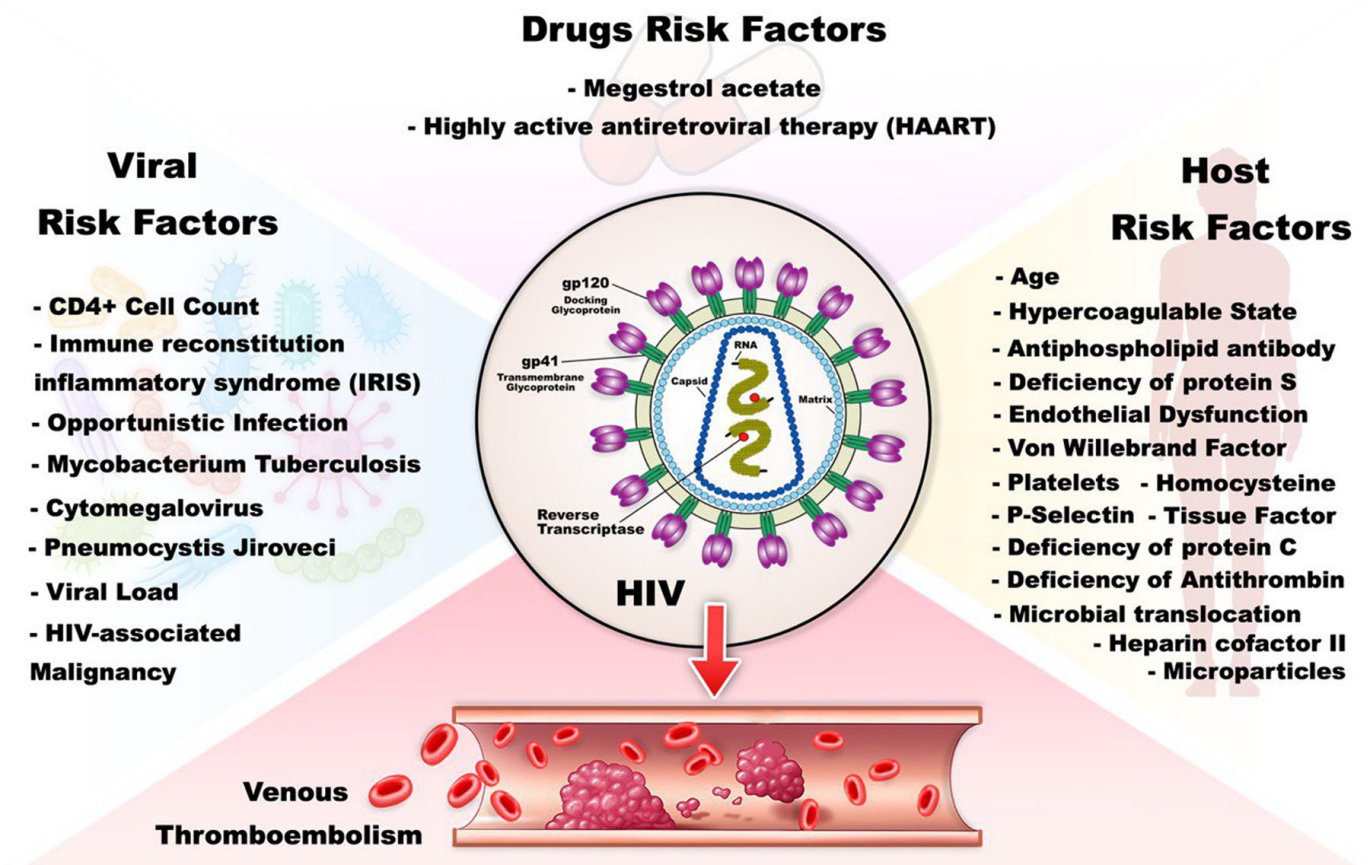
Venous thromboembolism (VTE) and HIV. VTE in HIV‐infected patients is divided into three main categories, including drugs risk factors, viral risk factors, and host risk factors.

Several studies recognized HIV infection as a prothrombotic condition.^5^, ^24^, ^34^, ^35^ The commonness of its occurrence in HIV patients with is 0.19%–7.63% per year.^3^, ^36^, ^37^ They also reported that the total elevated hazard of VTE in HIV patients is 2‐ to 10‐fold.^3^, ^36^, ^37^ Rasmussen et al.^3^ indicated that the 5‐year hazard of VTE is 1.5% in noninjecting drug users (non‐IDU) HIV patients and 8.0% in IDU HIV‐infected patients. The mechanism by which VTE occurs in HIV‐infected patients needs further study and still, less has been done on assessing the exact role of thrombo‐prophylaxis in patients with HIV.^1^ Many factors are linked with VTE in patients with HIV which are divided into three categories: (1) drugs risk factors, (2) viral risk factors, and (3) host risk factors^1^ (Figure 2).

### 3.1 Drugs risk factors

#### 3.1.1 Megestrol

Megestrol acetate is a progestational, orally active, and synthetic agent which is one of the treatments for breast cancer. In cachexia and/or anorexia patients with HIV, it stimulates weight gain and appetite. In HIV‐infected patients, thromboembolic phenomena occur as an unfavorable effect of megestrol.^38^, ^39^

#### 3.1.2 Highly active antiretroviral therapy (HAART)

HAART and especially protease inhibitors (PI) have been stated to be associated with VTE.^40^, ^41^, ^42^ PI interferes with hepatic metabolism, particularly the metabolism of cytochrome P450, and thrombotic proteins regulation. This can result in a prothrombotic condition in patients infected with HIV. This also can generate dysfunction in platelet or endothelial or down‐regulate the anticoagulant effects in the body.^43^ PI is associated with lipodystrophy. Patients infected by HIV with redistribution of fat can be at elevated hazard of advanced anomalous coagulation, including deficiency of protein S (PS), fibrinogen, plasminogen activator inhibitor‐1, or D‐dimer^44^ Indinavir and saquinavir are linked with VTE in HIV‐positive individuals.^40^, ^45^, ^46^, ^47^

### 3.2 Viral risk factors

#### 3.2.1 CD4^+^ cell count

HIV severity is significant in association with VTE. Low CD4 counts can cause an increased risk of VTE.^41^, ^48^, ^49^, ^50^ However, Crum‐Cianflone et al.^51^ established that thrombosis can happen with CD4 cell counts as 800 cells/mm3. This could recommend that thrombosis hazard is not overall limited to end‐stage disease. CD4 count and the risk for thromboses can be associated with the elevated hypercoagulable condition in the progression of HIV. Several studies indicated that impairment in procoagulant and anticoagulant factors can swing the pendulum in favor of thrombosis.^36^, ^52^, ^53^, ^54^

#### 3.2.2 Immune reconstitution inflammatory syndrome (IRIS)

IRIS is one of the general complications in patients who are beginning antiretroviral therapy, especially in patients who have experienced cryptococcal meningitis, tuberculosis, low CD4 cell counts, and cytomegalovirus retinitis.^55^ IRIS is characterized as a hyperinflammatory syndrome happening throughout immune recovery upon starting antiretrovirals.^56^ This can lead to a hypercoagulable state and VTE.^57^

### 3.3 Opportunistic infection

Despite HAART efficacy, HIV‐positive patients can develop opportunist infections relying on their immunologic condition. Therefore, opportunistic infections and developed HIV being concomitantly present can be an additional risk for VTE.^36^, ^43^, ^58^ VTE is mostly established with Mycobacterium avium intracellular, CMV, and pneumocystis jiroveci pneumonia (PCP).

## 4 INTRACELLULAR MYCOBACTERIUM AVIUM AND MYCOBACTERIUM TUBERCULOSIS

Mycobacterium‐avium intracellular and *Mycobacterium tuberculosis* can cause hypercoagulable conditions and anticardiolipin antibodies induction.^59^ After introducing the effective treatment for the underlying infection, the levels of anticardiolipin antibodies can decrease.^60^, ^61^ Mycobacterium tuberculosis activates macrophages and promotes the activation of cytokines, particularly interleukin‐6 (IL‐6), tumor necrosis factor‐alpha (TNF‐α), and IL‐1. In addition, IL‐1 and TNF‐α can suppress protein C anticoagulant pathways and evoke the production of tissue factors on monocytes and endothelium.^62^ IL‐6 stimulates the formation of new platelets. VTE prevalence in patients with Tuberculosis is 0.6%–3%. However, VTE prevalence in patients who have both HIV and tuberculosis is still unknown.^63^, ^64^

## 5 CYTOMEGALOVIRUS

CMV is a cause of thrombosis. Many studies reported active CMV infection in patients with VTE.^65^, ^66^ With the use of HAART, regarding patients with HIV, active CMV infection has been reduced significantly.^67^ The VTE incidence in HIV patients with CMV is estimated at 9.8%. Most thrombosis in these patients is a result of gastrointestinal disease.^41^

## 6 PNEUMOCYSTIS JIROVECI PNEUMONIA

Several studies indicated the presence of PCP throughout VTE in patients with HIV.^37^, ^43^, ^45^, ^68^ PCP‐associated VTE is secondary to the hypercoagulable condition in acquired immune deficiency syndrome (AIDS). PCP patients presenting with HIV can have elevated concurrent antiphospholipid syndrome which increases the risk of VTE.^69^

## 7 VIRAL LOAD

Viral load, also named HIV ribonucleic acid (RNA) level, is one of the indicators of the high disease burden of HIV. Decreased numbers of CD4^+^ cells and increased viral load scans predict HIV progression. Crum‐Cianflone et al.^51^ established that a lower number of CD4^+^ cells along with a higher viral load can increase the risk of thrombosis. However, some studies found no connection.^43^

## 8 MALIGNANCY ASSOCIATED WITH HIV

The risk for developing VTE in cancerous patients differs and is estimated to range from 15% to 30%.^70^ HIV and AIDS patients have an elevated risk of cancer.^71^ HIV‐infected populations have a 77‐fold elevated risk of non‐Hodgkin lymphomas (NHL), a six‐fold elevated risk of cervical cancer, and a 3640‐fold elevated risk of Kaposi sarcoma (KS).^72^ These malignancies are AIDS‐defining cancers.^73^ There is also an elevated risk for the formation of non‐AIDS‐defining cancers, including cancers related to co‐infections (e.g., Hodgkin lymphoma with the presence of EBV, anal and oropharyngeal cancers with the presence of human papillomavirus (HPV), and liver cancer with the presence of hepatitis B and C viruses).^74^ KS is one of the general malignancies correlated with VTE in HIV patients. The occurrence of thromboembolism in KS and HIV patients ranged from 9.3% to 20%. Other malignancies were rare including colon cancer, prostate cancer, B‐cell NHL, anal cancer, primary central nervous system (CNS) Lymphoma, and Hodgkin's disease.^45^, ^51^, ^58^

### 8.1 Host risk factors

#### 8.1.1 Age

Age is known to be a common risk agent for developing thrombosis. VTE incidence elevates as the population ages.^75^ Since most patients with HIV are young, the incidence of VTE is low. However, several studies indicated that the median age of patients with HIV who developed VTE is about 40 years old.^76^ It is younger than the median age by 20 years in individuals who are not infected.^41^, ^77^ Moreover, patients with the age of fewer than 50 years presented with HIV had an incredibly higher risk of VTE than the age‐matched healthy controls.^45^ Patients with HIV are older than their chronological age. They experience Premature Aging. In this state, even after treatment, the immune system has persistent defects. Many are the same as seen in normal age, however, they can happen at a younger age than the normal age.^78^ These persistent impairments are decreased vaccine responsiveness, CD28^−^ effector T cell expansion, low CD4:CD8 ratio, decreased T cell repertoire, and low naïve memory cell ratio. The majority of these impairments are observed only in patients who begin treatment in the late stage of the disease.^79^ The increased risk of premature aging is a cause of residual inflammation and immunodeficiency.^1^

#### 8.1.2 Hypercoagulable state

The hypercoagulable state is defined as a state where anticoagulant factors are reduced or procoagulant components are activated causing a hematological system that is at risk of thromboembolism. Many processes can affect the hypercoagulable condition in patients with HIV such as activation of D‐Dimer, C‐reactive protein (CRP), soluble TNF receptors, and IL‐6.^80^

#### 8.1.3 Antiphospholipid and lupus anticoagulant antibodies

Antiphospholipid syndrome (APS) is deemed an autoimmune disease. Anticardiolipin antibodies (ACA) along with lupus anticoagulant (LA) show up in these diseases. APS frequency is 2%–4% and it is associated with the hazard of developing venous and arterial thrombosis.^76^ ACA appears in patients with HIV with an incidence of 7%–94%.^81^ Several studies reported a positive connection between VTE and ACA in patients with HIV.^82^, ^83^ LA varies more in prevalence and manifestation in HIV patients. It has an incidence of 0%–72%. However, no pathogenic association was observed with thromboses in HIV patients.^82^, ^83^ LA effects can be epiphenomenon secondary to stimulation of the chronic immune in patients with HIV.^1^

#### 8.1.4 Deficiency of PS

The deficiency of PS is a result of HIV pro‐coagulant. Deficiency of PS is consistently seen in coagulation abnormality in patients with HIV with an incidence of 27%–76%, with 12% of those patients who are diagnosed with VTE.^42^, ^84^ The deficiency of PS in patients with HIV is multifactorial and alteration/deficiency frequency resulted in a wide pursuit of potential mechanisms. Type III deficiency of PS is one of the most general impairments observed and it is defined through the normal total level of PS with reduced functional PS activity and free PS.^85^

#### 8.1.5 Endothelial dysfunction

There is a strong correlation between VTE and endothelial cells abnormalities.^86^, ^87^, ^88^ In a normal state, endothelial cells can exert a local fibrinolytic, vasodilatory, and antiplatelet tone that can prevent coagulation of blood, adhesion of platelet, and attachment of leukocytes. Thrombomodulin production, heparan and dermatan sulfate expression, the fundamental activation of tissue factor signaling pathway inhibitor, and tissue plasminogen activator and urokinase‐type plasminogen activator production maintain nonthrombogenic endothelial surface. They are major physiologic fibrinolysis effectors. Nitric oxide and prostacyclin synthesis are important for the antithrombotic activities of the endothelium.^89^ In VTE, the dysfunctional venous endothelium can cause increased activation of plasminogen activator inhibitor‐1, von Willebrand factor, tissue factor, P‐selectin, along with factor V. They can induce clotting of blood and also participate in thrombus development.^90^, ^91^ HIV can directly activate the endothelium.^92^, ^93^

#### 8.1.6 Miscellaneous factors of hemostasis

In course of HIV‐1 infection, different kinds of endothelial cell damage markers including angiotensin‐converting enzyme, adhesion molecule E‐selectin, von Willebrand factor, fibronectin, soluble thrombomodulin, endothelin, tissue‐type plasminogen activator, plasminogen activator inhibitor, and fibronectin are elevated.^91^, ^92^, ^93^, ^94^ The deficiency of heparin cofactor (HC) II was incredibly more explicit in AIDS patients than in HIV patients. It could be due to the deficiency of HC II can be the enhanced consumption or proteolysis reduced synthesis.^95^ But, further studies are required to show the direct association between reduced levels of HC II in patients with HIV and VTE. Von Willebrand factor, soluble thrombomodulin, plasminogen activator inhibitor along with plasminogen activator secretion can cause alternation in the cascade of coagulation and result in thrombosis predisposition.^96^, ^97^ Moreover, HIV gp120 induces the expression of tissue factors in vascular smooth muscle cells, that can affect arterial wall thrombogenicity.^98^

#### 8.1.7 Von willebrand factor

Endothelial cell activation is associated with the release and also continuous bond to the local vessel wall of the von Willebrand factor. Many studies indicated increased von Willebrand factor levels in HIV patients.^99^

#### 8.1.8 Platelets

Platelets from patients with HIV are more frequently activated than controls.^100^ Chronic platelet activation from HIV patients is more correlated with the exhaustion of platelet granule content.^101^

#### 8.1.9 Homocysteine

In patients with HIV, Hyperhomocysteinemia ranged from mild to moderate, particularly those who used combination antiretroviral therapy (cART), with an incidence of 11%–29%.^52^, ^102^ The raise in recurrence risk correlated with increased concentrations of homocysteine is about 1‐ to 5‐fold.^76^, ^103^ But, since vitamin supplementation, which decreases concentrations of homocysteine, does not have any effects on the recurrence rate, the association between VTE and hyperhomocysteinemia remains unknown.^56^ Hyperhomocysteinemia is mostly present in patients with HIV without any clinical manifestation indicating that it may not be enough to cause VTE.^1^

#### 8.1.10 P‐selectin

P‐selectin in HIV‐infected individuals is correlated with VTE.^104^ Stored in platelet granules and endothelial cells, P‐selectin can interact with its receptor to induce hypercoagulation via activation of tissue factor on monocytes and promoting prothrombotic microparticle generation from leukocytes.^105^ P‐selectin is incredibly increased in imminent or acute VTE. Moreover, P‐selectin and D dimer in VTE patients are alike in terms of diagnostic value.^106^

#### 8.1.11 Tissue factor (TF)

Funderburg et al.^60^ established increased prevalence in monocytes that express TF in fresh blood samples from patients with HIV. They hypothesized that different kinds of bacterial toll‐like receptor (TLR) ligands, including flagellins, peptidoglycans, and lipopolysaccharide, are translocated via the injured gut in chronic HIV and can cause activation of the immune and expression of monocyte TF. The correlation between elevated expressions of TF in HIV is underlined by the association between levels of D‐dimer and expression of TF and by the increased D‐dimer levels in plasma. Replication of HIV and the microbial products systemic translocation of the injured gut, and further activation of the immune, associated with a procoagulant condition in patients with HIV that is partly due to elevated TF surface expression on circulating monocytes.^107^

#### 8.1.12 Protein C (PC) deficiency

The correlation between PC deficiency and VTE is not completely defined with an incidence of 0%–14%. However, Majluf‐Cruz et al.^108^ observed a high incidence of deficiency of PC in HIV patients who developed VTE. PC shortage mechanism in HIV involves a number of factors, including altered metabolism, altered synthesis, and consumptive coagulopathy.^48^

#### 8.1.13 Antithrombin deficiency

There are not enough studies to indicate that HIV leads to the deficiency of antithrombin. Acquired deficiency of antithrombin happens during HIV due to correlated conditions which lead to reduced protein synthesis, consumptive states, as well as protein‐losing enteropathies or nephropathies. Several studies reported the deficiency of antithrombin in patients with HIV who had thromboses.^43^, ^109^, ^110^

#### 8.1.14 Heparin cofactor II (HC II)

HC II deficiency has the potential to be a risk factor for thrombosis. Heparin cofactor II shortage is incredibly greater in HIV patients. Their correlation was explained by the absolute number of CD4^+^ T lymphocytes as well as the CD4/CD8 ratio. Shortage of heparin cofactor II is more significant in patients with AIDS in comparison to patients with HIV.^95^

#### 8.1.15 Microparticles

Microparticles mean small membrane vesicles which are released from apoptotic CD4^+^ lymphocytes, endothelial cells, platelets, vascular smooth muscle cells, as well as tumor cells.^50^ In healthy persons, reduced microparticles are current in plasma with plasma free. However, increased levels have been found in patients with HIV, but it is unknown that this can result in increased VTE risk.^111^

#### 8.1.16 Translocation of microbes

The translocation of microbes is the main characteristic of the pathogenesis of HIV and AIDS. Early damage in the gastrointestinal tract of patients with HIV causes microbial products systemic translocation, such as LPS and bacterial DNA.^112^ Selected TLR ligands promote the expression of TF on monocytes in the blood. Soluble TF levels elevate in patients with HIV and monocyte levels are associated with LPS coreceptor sCD14 along with the plasma levels of D‐dimers. LPS exposure can promote P‐selectin expression in platelets.^113^ The bacterial product recognition causes expression of TF and activation of monocytes, causing elevated monocyte TF expression and production of pro‐inflammatory cytokine.^114^

#### 8.1.17 Intravenous drug use

The intravenous recreational drugs use is correlated with significant morbidity.^115^ IDU is a crucial reason for community‐acquired VTE in young adults.^116^ It has been established the effects of IDU on VTE in patients with HIV. They showed that the hazard of VTE is 15‐fold higher in IDU patients with HIV than non‐IDU HIV patients.^1^, ^3^

### 8.2 Risk factors related to treatment

#### 8.2.1 Antiretrovirals

Howard et al.^117^ found no correlation between antiretrovirals and VTE. However, it has been observed in adjusted models that integrase inhibitor use (raltegravir) correlates with the total VTE risk, based on statistical evidence.

## 9 MANIFESTATION OF UNUSUAL THROMBOSES

Development of thrombosis at anomalous sites is a cause for almost 10% of the entire VTE cases, impacting the venous region except for superficial or deep veins in the lower limbs or veins of the pulmonary circulation. For retinal thrombosis, its prevalence is 5 per 1000 individuals, and for splanchnic vein thrombotic events, its incidence is 1–2 cases for every 1 million people.^118^

## 10 OCCLUSION OF RETINAL VEIN

Occlusion of the retinal vein involves the branch, central or more seldom, the Hemi central vein. It mostly occurs upon glaucoma, intraocular hypertension, or via the systemic risk factors of arterial thrombotic events. Venous occlusions and retinal arterial in patients with HIV are commonly correlated with lymphoproliferative disorders and CMV retinitis.^119^ Occlusion of the central retinal vein happens in 0.64% of AIDS patients.^120^ Dunn et al.^121^ reported that occlusion of the retinal vein was observed in 38 eyes of 33 (1.3%) of the 2484 patients. Furthermore, HIV patients have a low chance to develop occlusion of the retinal vein, however, visual loss and complications are more general in patients with HIV.^114^

## 11 THROMBOSES IN CEREBRAL VENOUS SINUS

Thromboses in the cerebral venous sinus in the normal population are common among young women due to their strong relationship to the use of oral contraceptives and pregnancy. In HIV patients, the clinical course is milder in comparison to the normal population. Elevated risk of thrombosis as a result of Vitamin B12 deficiency and increased homocysteine.^122^

## 12 THROMBOPROPHYLAXIS

Throughout pregnancy and in HIV patients, presented with a malignant disease, the incidence of VTE indicated respectively an upper limit of the 95% confidence interval of 17.3 and 25.1 for every 1000 person‐years.^117^ But, this does not go beyond the threshold rate of oncological guidelines (about 10% 1‐year rate or 100 per 1000) and the threshold incidence rate of the American College of Chest Physicians Guidelines (20 per 1000).^123^, ^124^ However, not using thromboprophylaxis is general in patients with HIV. Diagnosis of HIV is incredibly correlated with documented dose refusal and dose nonadministration, being about two times higher for HIV patients in comparison to non‐HIV patients.^125^ Furthermore, since HIV patients are at elevated VTE development risk, making sure that thromboprophylaxis is delivered efficiently and safely could be a crucial aim in HIV patients.^114^

## 13 PREGNANCY PUERPERIUM

The incidence of VTE in pregnant women is nearly 1–2 per 1000 pregnancies. Pregnant women can develop VTE 5‐fold greater than nonpregnant women.^1^ Pham et al.^126^ showed that VTE annual prevalence in women with HIV throughout puerperium is 313 for every 1000 person‐years. Their result indicated that HIV‐infected women during pregnancy develop VTE 120‐fold more than controls infected by HIV. However, the risk is 157 times higher in comparison to pregnant women with negative HIV.^1^ In patients with HIV, the frequency rate of the first thrombotic incident throughout pregnancy was established as 9.4 for every 1000 person‐years.^117^

## 14 CLINICAL PRESENTATION

Clinicians should be aware of unprovoked thrombosis. It is one of the AIDS complications and it should be considered in the various diagnosis of HIV‐infected patients. It can be possible that thromboembolic disease incidence could be underestimated owing to not accounting for the less established complication including VTE or mimicking opportunistic infections.^127^ Thrombosis clinical appearance and distribution are similar to non‐HIV individuals.^43^, ^45^, ^51^ Thromboses commonly occur in popliteal and femoral veins followed by pulmonary emboli.^37^, ^128^ Moreover, involvement of the abdomen can happen as splenic or portal vein thromboses.^129^, ^130^ HIV‐infected individuals can experience recurrences with a rate of 8%–15%.^41^

## 15 MANAGEMENT

Clinicians who are dealing with HIV should be alert that HIV patients require significant attention regarding antithrombotic prophylaxis and therapy. Its management is the same as patients without HIV, such as long‐term use of prophylaxis with LMWH as well as warfarin with recurrent thrombotic events.^1^

### 15.1 Antithrombotic therapy in HIV

#### 15.1.1 Warfarin with antiretrovirals

The hazard of developing bleeding or stroke through the use of warfarin as a treatment is straightly associated with the time proportion within the international normalized ratio (INR) in the scope of therapy. Patients with HIV have a lower therapeutic range in comparison to the general population. Drug‐drug interactions can cause difficulty to attain therapeutic range, and the reason for this interaction is warfarin metabolism alternation via elevation or reduction in the activity of cytochrome P450 2C9 enzyme. Requirements of warfarin dose significantly elevate in patients introducing boosted PI and nevirapine. However, efavirenz is correlated with the development of overdosing on warfarin and bleeding. Moreover, the use of etravirine can result in the inhibition of warfarin metabolism. The use of warfarin is found to not have interaction with maraviroc or NRTI. Amongst INSTI, elvitegravir solely affects cytochrome P450 isoenzymes by promoting the activity of CYP2C9. Since it is co‐formulated with cobicistat, mild opposite influences can be observed. However, raltegravir can be introduced with warfarin securely.^131^, ^132^, ^133^

#### 15.1.2 Direct oral anticoagulants (DOACs) with antiretrovirals

Oliveira et al.^134^ reported that DOACs is more efficient than conventional anticoagulant. CYP3A4 along with P‐glycoprotein transporter (P‐GP) can metabolize DOACs.^114^

#### 15.1.3 Integrase inhibitors

Elvitegravir enhanced with ritonavir or cobicistat is commonly not suggested whilst using DOACs.^114^

#### 15.1.4 Nonnucleoside reverse transcriptase inhibitors

Efavirenz, etravirine, as well as nevirapine, can promote CYP3A4 causing DOACs to lose their anticoagulant impact.^123^ Concurrent nevirapine can elevate rivaroxaban clearance, resulting in PE. Rilpivirine and etravirine (Weak P‐GP inhibitors) can be potentiators of the anticoagulant impact of DOACs.^135^ Since nevirapine or efavirenz cannot impact P‐GP, with the deficiency of induction/inhibition of cytochrome P450 isoenzymes, no interplays have been reported.^114^

#### 15.1.5 Protease inhibitors (PIs)

Coadministration of the anticoagulant impact of factor X‐activated inhibitors and strong inhibitors of P‐GP and CYP3A4 (including cobicistat and ritonavir) can result in bleeding events.^136^ PIs are inhibitors in a lesser amount except for rivaroxaban. Corallo et al.^137^ showed bleeding events happen with the coadministration of ritonavir‐boosted darunavir and rivaroxaban. Since dabigatran has a unique metabolism amongst DOACs, it can be used with antiviral therapies. Among PIs no substantial interaction was observed while dabigatran was concurrently used with ritonavir in healthy controls, regardless of being one of the strong P‐GP inhibitors. Administration of concurrent cobicistat considerably elevated dabigatran anticoagulant impact.^138^

#### 15.1.6 Other antiretrovirals

Maraviroc and nucleoside reverse transcriptase inhibitors (NRTIs) do not impact the metabolism of DOACs and can be administered simultaneously without any interactions being expected. It is suggested to preclude factor X‐activated inhibitors, such as apixaban and rivaroxaban combined with non‐NRTIs, cobicistat, or PIs.^123^, ^135^

#### 15.1.7 Risk of developing recurrent VTE in HIV patients

Reports indicate that HIV patients are at a greater hazard of developing recurrent VTE in comparison to controls uninfected by HIV.^30^ VTE relapsed more commonly in men as well as the first year after ceasing the use of anticoagulants. Moreover, the recurrence risk was reduced in patients with HIV with better immune recuperation over time and also increased CD4^+^ T, cell numbers. HIV patients who have an unstimulated first VTE along with significant recovery of CD4^+^ T cells throughout anticoagulation therapy are at low risk for VTE. In conclusion, hypercoagulable state complexity is because of HIV‐related immunodeficiency as a risk for VTE that is capable of being reversed.^139^ The higher the CD4^+^ T‐cell number recovery the lesser risk of developing venous thromboembolism.^114^

### 15.2 CMV and VTE

#### 15.2.1 Epidemiology

CMV infection has a seroprevalence of about 60%–100% depending on different factors such as age, socioeconomic, country, and conditions.^140^ Since 1974 Vorlicky et al. hypothesized the possibility of a connection between CMV infection and VTE,^141^ and this has been reported several times in the literature since 1984^142^ the incidence of VTE is reported to be greater in CMV seropositive persons than in CMV seronegative persons up to 6 months from the index date.^143^ CMV seropositivity is also associated with arterial thrombosis but is less convincing, with an odds ratio of 1.23.^144^, ^145^ according to the study by Atzmony et al.,^146^ the acute CMV infection‐associated thrombosis incidence amongst hospitalized patients was 6.4%: five (3.6%) patients who had arterial thrombosis and four (2.9%) patients who had VTE. However, the true thrombosis incidence is probably higher. The thrombosis incidence amongst out‐patients following acute CMV infection has been investigated once by Paran et al.^143^ According to this study, the VTE incidence among out^147^ hospitalized thrombosis patients has been studied prospectively twice by Tichelaar et al.^148^ and Schimanski et al.,^149^ which respectively showed a 1.9% (*n* = 5) and 4.3% prevalence.

#### 15.2.2 Pathophysiology

Different mechanisms describe the formation of VTE in CMV‐infected patients. Herpesviruses envelope procoagulant phospholipids (pro‐PL), and tissue factor (TF) can initiate the formation of thrombin.^150^, ^151^ Endothelial cells infected by CMV upregulate the expression of VCAM‐1 and ICAM‐1 as adhesion molecules^152^ and TF on their surfaces. Hence, it leads to platelet aggregation on vessel walls,^153^ thrombin formation,^154^, ^155^ and factor X activation. In another word, the infection of the endothelial cells leads to the release of TF‐mediated thrombin, competition, reduction of anticoagulants, and inhibition of the fibrinolysis.^156^ It has been suggested that CMV may increase circulatory levels of von Willebrand factor and factor VIII.^157^, ^158^ Currently, the most accepted theory as the major mechanism for thrombosis formation in CMV infection is the transient hypercoagulable state which occurs due to the production of APS and anticardiolipin antibodies.^83^ However, by the time the infection abates, the levels of these antibodies decrease.^125^, ^147^, ^159^, ^160^ However, as Uthman et al.^161^ stated in their study antiphospholipid antibody positivity is not specific to CMV infection, and other conditions can stimulate the formation of this antibody, such as syphilis and many other viral infections. However, there is something unique about CMV, and that is a true transient antiphospholipid antibody syndrome.^160^ Although each of the mechanisms above might bring about this procoagulant state, the impact seems to be impermanent as the anticardiolipin antibodies clear by months passes from the CMV infection.^142^ Furthermore, the CMV viremia and hepatitis combination caused by CMV probably come up with the procoagulant state, resulting in portal vein thrombosis.^154^, ^162^ Some suggest that secondary CMV infection seems to be more thrombogenic than primary infection,^149^ although most of the time it is impractical to determine whether it was primary or secondary.^142^, ^163^, ^164^

#### 15.2.3 Symptoms

Acute CMV infection normally appears asymptomatic in immunocompetent patients or may characterize by mild flu or a few other symptoms, including mononucleosis‐like syndrome.^26^ However, suppression of the immune system allows CMV infection to manifest by more severe symptoms including pneumonia, hepatitis, pericarditis, myocarditis, colitis, hemolytic anemia, thrombocytopenia, and vascular thrombosis are reported.^146^, ^162^, ^165^ Among these, mononucleosis and hepatitis have the highest prevalence (*n* = 76; 67.3%). CMV colitis comes after that (*n* = 10; 8.8%). Other manifestations include retinitis (*n* = 5; 4.4%), Guillain‐Barré syndrome (*n* = 1; 0.9%), pneumonitis (*n* = 1; 0.9%), encephalitis (*n* = 1; 0.9%), and (5.3%) patients had no clinical signs of CMV disease and were detected in the course of unknown origin fever workup by serology tests or viremia markers.^142^, ^163^, ^164^

#### 15.2.4 Sites of thrombotic involvement

CMV‐related thrombosis appears to involve large vessels as well as small ones, arteries, along with veins, making it challenging to anticipate its effects. However, arterial thromboses linked with acute CMV infection have been rarely reported in the literature.^166^ CMV‐associated thrombosis involves different sites, including the lower limbs and pulmonary arteries, which respectively leads to DVT and PE.^142^ Splanchnic vein thrombosis (SVT), which involves portal vein thrombosis, mesenteric vein thrombosis, and splenic vein thrombosis, along with the Budd‐Chiari syndrome, is also reported to be affected.^167^ The most common site involved was the pulmonary arteries, and The second most involved zone was the portal region,^27^ both of which are usual sites of thrombosis in an immunocompetent patient^26^, ^27^, ^158^; however, Justo and colleagues reported that lower limb DVT and PE are the most frequent thrombotic complications of CMV mononucleosis, with splanchnic thromboses accounting for the second‐highest prevalence.^142^ DVT and PE are more prevalent amongst immunocompromised patients whereas splanchnic vein thrombosis is more prevalent amongst immunocompetent patients.^142^

#### 15.2.5 Treatment

Despite being a severe complication, CMV‐related VTE appears to be benign in adults, and most of the time, we can see a complete resolution; however, newborns and infants seem to have a poor prognosis in comparison to adults.^27^ There is no agreement about the administration of antiviral and anticoagulant therapy in patients who are immunocompetent, also the consumption of this treatment in these patients is off‐label. besides, its usefulness is not proved.^27^ Overall, 30.1% of patients whom most of them had viremia, and have been treated with antivirals, that is, ganciclovir and/or valganciclovir,^21^, ^164^, ^168^, ^169^ Antiviral Agents have been used in Immunocompromised patients more than immunocompetent patients.^142^ Some researchers^27^, ^170^ have reported that they did not apply anticoagulation therapy in patients with CMV‐associated splenic infarcts; however, their patients also had coagulation disorders, such as heterozygous factor V Leiden. In the mentioned condition it is suggested to use anticoagulant therapy for about 6–12 months after the first episode of VTE, primarily if related to impermanent risk factors such as the CMV infection.^171^, ^172^, ^173^

In some reports, anticoagulation therapy has been continued until the anti‐phospholipid antibodies disappeared or the thrombosis is resolved in imaging studies.^21^, ^174^ One prospective study described 6 months gap postinfection when the rate of VTE is higher in comparison to the control population,^143^ This is especially of great importance for the setting of the 2016 VTE guidelines, which attract attention to chronic anticoagulation therapy in every patient with recent, unprovoked VTE.^175^ A systematic review and meta‐analysis investigated the function of anticoagulant and antiviral therapy in VTE cases in CMV‐seropositive immunocompetent adults.^176^ The authors concluded that the antiviral medications’ function in this examination remains unknown, as the desired results might be imputable to the self‐limited nature of CMV infection. It is recommended to keep an eye on bone marrow toxicity while using antiviral medications.^145^ For anticoagulant therapy, the patients received medication based on guidelines for PE and thrombosis. For antiviral therapy, the patients received valganciclovir and/or ganciclovir for about 3 weeks, and Both groups attained good outcomes.^172^

#### 15.2.6 Risk factors

Predisposing factors for CMV‐associated thrombosis are more significant among immunocompetent patients than immunocompatible ones.^142^ There are two main categories of risk factors for CMV‐associated thrombosis. Modifiable ones include Estrogen–progestogen contraception which can be taken orally or intravaginal,^177^, ^178^, ^179^, ^180^, ^181^ smoking, pregnancy as well as hypertension, previous VTE, and cancer. The last three factors are well‐known for thromboembolic events. Pregnancy elevates the hazard of VTE 4–5 times over,^182^ non‐modifiable risk factors include heterozygotic Factor V Leiden, heterozygotic factor VIII, heterozygosis for the G20210A prothrombin mutation, MTHFR mutation, Protein C deficiency, lack of PS, hyperhomocysteinemia. The patients’ blood samples displayed a transient increase of some autoantibodies such as antiphospholipids, autoantibodies, anticardiolipin antibodies, and antinuclear antibodies along with LAC.^27^

#### 15.2.7 Diagnosis

Diagnostic methods for CMV infection in patients with thrombosis include serologic testing, the presence of CMV antigens in urine, epithelial cell inclusion bodies on tissue biopsy, antigenemia, and viral load testing. According to a study by Paran et al., positive serology for CMV IgM is the modest independent predictor of VTE occurrence (odds ratio of 2.49 for VTE as a whole and 2.44 for PE or infarction and lower limb DVT).^145^ It has been suggested to confirm the CMV infection with proper viral load testing (not static serology in isolation) as CMV IgM tends to stay positive for weeks to months after the acute infection.^144^, ^145^, ^183^

#### 15.2.8 Mortality

Five (4.4%) death have been reported in general, all of which were immunocompromised.^146^, ^184^, ^185^, ^186^ The mortality rate is probably higher as there might be biased reports. As reported by Atzmony et al.,^170^ in‐hospital mortality rates amongst CMV‐associated thrombosis patients are 22.2%, but long‐term and also out‐of‐hospital mortality have never been documented in these patients.^21^

#### 15.2.9 Zika and Chikungunya virus and VTE

The Zika and Chikungunya viral infections are spread by different species of Aedes mosquitoes. Fever, headache, arthralgia, myalgia, along with a maculopapular rash are characteristic of both diseases, and studies have linked Zika infection with microcephaly in newborns.^6^

Ramacciotti et al. Studied patients displaying symptoms of Zika and Chikungunya infections, once the reports of DVT were confirmed, blood samples were prospectively taken for D‐dimer analysis with confirmed viral Zika and Chikungunya infections according to polymerase chain reaction tests. As a result, D‐dimer levels were higher than the normal scope in 19.4% of 31 Zika patients and 63.8% of 141 Chikungunya patients.^6^

Therefore, it has been shown that Zika and Chikungunya may increase the hazard of clinical VTE, as indicated by elevated D‐dimer levels, but there is no sufficient evidence to support the link between Zika or Chikungunya and VTE yet.^6^

#### 15.2.10 Epstein–Barr virus and VTE

Infection caused by EBV is mostly asymptomatic or related to flu‐like symptoms. But sometimes it is associated with mononucleosis syndrome, hepatosplenomegaly, cervical lymphadenopathy, pharyngitis, hepatitis, monocytosis, and atypical lymphocytosis. VTE caused by EBV is mostly reported in immunocompromised patients and some in patients with antiphospholipid antibody syndrome.

Monocytosis, elevation in erythrocyte sedimentation rate (ESR) and CRP serum levels, normal protein C and S levels, normal C3 levels, and elevated C4 levels were seen in these patients. In addition, anticardiolipin antibodies and rheumatoid factor were negative. Antithrombin therapy (Warfarin) was administered to patients presenting with VTE caused by EBV.^187^

Bader et al.^13^ described a 61 years old man that developed VTE after an EBV infection. EBV causes infection in more than 95% of humans as they grow older. The virus causes infectious mononucleosis (IM) which is characterized by fever, tonsillar pharyngitis, and lymphadenopathy triad. EBV can cause rare situations like acute interstitial nephritis, encephalitis, hepatitis, myocarditis, hemolytic anemia, disseminated intravascular coagulation (DIC), and thrombocytopenia.

Anti‐VCA IgG, anti‐VCA IgM, anti‐EBNA IgG, and EBV plasma viral load were all detectable and alanine aminotransferase, aspartate aminotransferase, as well as lactate dehydrogenase, were elevated. Endothelial cell inflammation, pro‐inflammatory cytokines, elevations of antiphospholipid antibodies, EBV is also linked with certain malignancies including lymphoma that may result in thrombosis, and EBV‐induced DIC is a mechanism that may potentially cause a procoagulant state along with the formation of thrombosis. Subcutaneous unfractionated heparin was replaced with graduated compression stockings for the aim of prophylaxis of the VTE.^13^

#### 15.2.11 Varicella‐Zoster virus and VTE

VZV is from the Alpha‐herpesvirus subfamily, there are two different types of disease associated with VZV‐caused infection: chickenpox and shingles. The virus is associated with different neurological problems like encephalitis, meningitis, ventriculitis, cerebellar ataxia, ischemic or hemorrhagic, and cerebral venous sinus thrombosis (CVST).

In the patient observed in this study varicella zoster IgG and IgM antibodies were positive also the patient had transient seizures after admission. Congenital thrombophilia, oral contraceptive usage, autoimmune diseases, malignancies, and infections are risk factors of CVST. VTE in VZV is caused by antiphospholipid antibody production. The patient was treated with acyclovir, anticoagulation, and levetiracetam. Prompt recognition and treatment are important to prevent catastrophic complications.^188^

In a study made by Ferrara et al.^14^ some patients showed VTE characterized by DVT. Primary thrombophilic situations were because of lower qualitative or quantitative antithrombotic proteins, and children infected with VZV with VTE had more intense responses compared to children without VTE, in these children genetic mutations at nucleotide position, and Prothrombin were also considered. It was observed that 20% of children who have DVT, develop post‐thrombotic syndrome as well as chronic venous insufficiency. Children were treated with heparin after special tests.^14^

Mathew et al. reported a 33‐year‐old married lady infected with VZV who had vesiculopapular rash, severe headache, vomiting, bilateral papilledema, and thrombosis in the right transverse, sigmoid, and straight sinuses. She was treated with acyclovir and dabigatran (anticoagulation).^189^

Symptoms caused by infection with VZV are Varicella lesions, fever, aseptic meningitis, encephalitis, pyomyositis, necrotizing fasciitis, purpura fulminans, cellulitis, and subsequent invasive bacterial disease, that may cause thrombotic initiation. The transient immune response consists of lupus anticoagulants, acquired antibodies against antithrombin, cardiolipin, and proteins C and S. Treatment was with intravenous flucloxacillin and clindamycin, anticoagulant treatment with subcutaneous enoxaparin, and then flucloxacillin was switched to ampicillin.^29^

#### 15.2.12 Hepatitis C virus and VTE

Two different studies by Enger et al. and Wang et al. concluded that there is a higher risk of thromboembolic incidents in patients diagnosed with HCV as well as cirrhosis compared to non‐HCV patients.^11^, ^12^ Furthermore, we must follow up on the cardiovascular disease in these patients, but thrombosis in veins (DVT, PE, and PVT) are more common such as thrombosis of the portal vein. Additionally, the Kaplan–Meier curve demonstrated that the incidence of DVT was higher in HCV infection.

DVT caused by HCV infection is caused by different reasons: (1) vasculopathy caused by chronic inflammation, (2) anticardiolipin and antiphospholipid antibodies presence, (3) Downregulation of anticoagulant proteins and upregulation of procoagulant proteins, (4) higher thrombin generation rates in patients with HCV and cirrhosis, and (5) higher cryoglobulinemia in HCV patients which can cause thrombotic vasculitis.

Finally, HCV infection is related to DVT in a long‐term follow‐up, and even patients with chronic HCV infection who are not diagnosed with liver cirrhosis may still have a higher risk of DVT.^11^, ^12^

#### 15.2.13 Herpes simplex and VTE

HSV is a neurotropic virus that infects peripheral sensory neurons and the central nervous system. HSV‐caused encephalitis and cerebral venous thrombosis connection are rare. Altered mental status, seizures, fever, headache, and focal neurological deficit are the common symptoms.

HSV ceases anticoagulant functions and causes a procoagulant phenotype. The virus reduces the synthesis of heparan sulfate proteoglycan. HSV infection increases the thrombin generation and platelet binding decreases prostacyclin synthesis and prevents antithrombin III binding to its surface. In addition, thrombomodulin expression is reduced, which affects protein C activation. The other mechanism is that HSV causes upregulation in endothelial binding sites for inflammatory cells and platelets, which causes the release of inflammatory cytokines. Management of encephalitis and cerebral venous thrombosis requires early treatment. Therapy is with acyclovir and anticoagulation, and the clinical improvement seropositivity prevalence is more than 60%.^9^

In a study by Marchalik et al., they considered a patient with pemphigus vegetans (PV) and HSV who developed DVT and septic shock. Treatment was with anticoagulation drugs, high‐dose prednisone, broad‐spectrum antibiotics, anti‐fungal drugs, acyclovir, and aggressive fluid resuscitation. It was seen that Pemphigus is linked with a greater incidence of thromboembolism and PV is linked to HSV.^8^

#### 15.2.14 COVID‐19 and VTE

**Figure 3:**
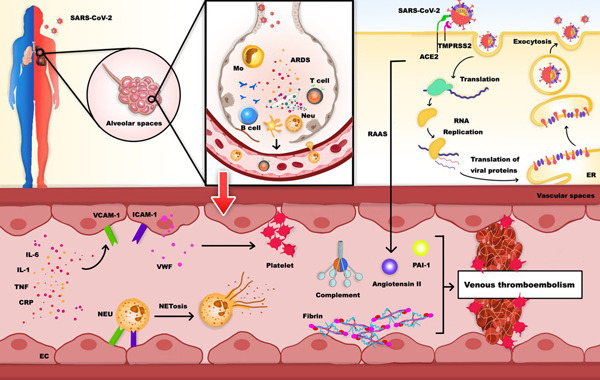
Venous thromboembolism and coronavirus. Severe acute respiratory syndrome coronavirus 2 (SARS‐CoV‐2) induces acute respiratory distress syndrome (ARDS), a type of respiratory failure characterized by rapid secretion of pro‐inflammatory cytokines and immune cells infiltrations in the alveolar spaces. ARDS can activate the fibrinolytic system. Besides, elevated fibrin deposition inside the vascular and alveolar spaces is caused by increased levels of PAI‐1 in ARDS. SARS‐CoV‐2 infection may reduce ACE2 activity which will end up in decreased angiotensin 1–7 and increased angiotensin II, which has prothrombotic and pro‐inflammatory impacts. Pro‐inflammatory cytokine can stimulate endothelial cells (EC) to secrete Von Willebrand factor (VWF) and increase the expression of adhesion molecules ICAM‐1 and VCAM‐1, resulting in the recruitment of more immune cells. Moreover, complement activation and NETosis can boost the immunothrombosis in the vascular system.

COVID‐19 resulted from the SARS‐CoV‐2 infection that began in Wuhan city of China in December 2019. SARS‐CoV‐2 is a novel Coronaviridae ribonucleic acid virus strain that was initially reported by Zhou and coworkers in February of 2020.^190^ The World Health Organization (WHO) classified COVID‐19 as a global pandemic in March 2020, affecting more than 100 nations by the end of May, with the number of new cases and fatalities quickly growing every day. The 157th Situation Report of the WHO on COVID‐19, released on June 25, 2020, revealed that the virus had infected over 9 million individuals globally, resulting in around 480,000 fatalities in only 6 months.^191^ Coagulopathy is linked to a variety of viral diseases, including HIV, CMV, Ebola–Marburg virus, VZV, Middle East respiratory syndrome CoV, and H1N1 influenza A virus. SARS‐CoV has all been linked to coagulopathy abnormalities such as disseminated intravascular coagulopathy, pulmonary thromboembolic, and DVT.^192^, ^193^ COVID‐19 has also been linked to severe coagulopathy, resulting in the development of a novel condition defined as COVID‐19‐associated coagulopathy, which affects the arterial, venous, and microcirculatory systems.^194^, ^195^, ^196^ It is known to induce natural anticoagulant dysregulation, resulting in platelet dysfunction and higher D‐dimer levels, as well as possible hyperactivation through inflammatory cascades.^194^, ^195^, ^196^, ^197^ with thromboelastograms of whole blood demonstrating heightened coagulation parameters.^198^ (Figure 3). In the following we will review some important topics:

#### 15.2.15 Fibrinolysis

Acute respiratory distress syndrome (ARDS) activates the fibrinolytic system.^199^, ^200^, ^201^ Elevated fibrin deposition inside the vascular and alveolar spaces is caused by increased levels of plasminogen activator inhibitor 1 (PAI‐1) in ARDS. In individuals with ARDS, high levels of plasma PAI‐1 are linked to death.^202^, ^203^ According to one study, 16 individuals with SARS‐CoV had a greater level of plasma PAI‐1 compared to 19 individuals with different infectious cases of pneumonia and healthy controls.^204^ In SARS‐CoV–infected mice, the expression of PAI‐1 was elevated, and PAI‐1/animals had more lung bleeding and died more often,^205^ inhibition of fibrinolysis by PAI‐1 seems to protect against intrapulmonary bleeding, according to this research. The application of thrombolytic medications to treat COVID‐19 was reviewed in a recent study that outlined the fibrinolytic anomalies related to ARDS. In individuals with COVID‐19, nebulized plasminogen activators were suggested as a way to break down alveolar fibrin and increase oxygenation.^206^ Recent research found that intravenous tissue plasminogen activator restored the respiratory status of three individuals with acute COVID‐19 respiratory distress for a short period.^207^

#### 15.2.16 Platelets

Platelets are necessary for arterial integrity, yet they can contribute to thrombosis. Platelets have recently been discovered to take part in the immunological response to infections.^208^ Influenza A virus (IAV) particles were found in platelets from individuals with acute influenza virus infection, which was surprising. In addition, platelet engulfment of IAV resulted in toll‐like receptor 7 (TLR7)‐dependent C3 release, neutrophil activation, as well as the release of neutrophil extracellular trap (NET).^209^ Platelets, consequently, have a role in the host's response to IAV infection. Platelet activation with a viral infection, on the other hand, may raise the hazard of venous thrombosis. In one research on COVID‐19 individuals, thrombocytopenia was linked to an enhanced risk of in‐hospital death.^210^ In recent research, the possible function of platelets in thrombosis in COVID‐19 was examined.^211^

### 15.3 Dysregulated renin‐angiotensin‐aldosterone system and the role of angiotensin‐converting enzyme 2 (ACE2)

A significant role of ACE2 is inactivating angiotensin II by transforming it into angiotensin 1–7 in a proteolytic way.^212^ This, therefore, puts ACE2 in a critical situation as the renin‐angiotensin‐aldosterone system (RAAS) negative regulator SARS‐CoV2 enters cells of humans via binding to the ACE2 by its spike protein (S‐protein),^213^ and as a result, may reduce ACE2 activity which will end up in decreased angiotensin 1–7 and increased angiotensin II.^214^ It is worth bearing in mind that whereas angiotensin 1–7 is acknowledged as a significant anti‐thrombotic and also an anti‐inflammatory peptide, angiotensin II has prothrombotic and pro‐inflammatory impacts inhibition of platelets activation is a result of increased nitric oxide and prostacyclin production via binding Angiotensin 1–7 to Mas receptors on the endothelium.^215^ ACE2's wide expression on endothelial cells has been displayed thus there is a possibility for direct SARS‐CoV 2 infection of the endothelial.^216^ Thus, dysregulation of RAAS in COVID‐19 patients’ vasculature system may trigger a cascade of incidents associated with enhanced coagulopathy which includes endothelial dysfunction, oxidative stress damage, and activation of von Willebrand factor.^214^

#### 15.3.1 Endothelium activation

The endothelium preserves vascular integrity, restricts immune cell as well as platelet binding and activation, and suppresses coagulation by expressing anticoagulant proteins under normal circumstances. The endothelium gets active during infection, bringing about a reduction in barrier function, the production of adhesion proteins that aid immune cell recruitment, the von Willebrand factor release that facilitates platelet binding, and TF expression that stimulates the coagulation system. In one research, soluble intercellular adhesion molecule 1 (ICAM‐1) and soluble vascular cell adhesion molecule (VCAM‐1) were linked to illness development in IAV (H1N1) pdm09 individuals who were hospitalized.^217^ These indicators suggest that circulating inflammatory mediators may activate the endothelium. Only IAV H5N1 from avian has been demonstrated to infect pulmonary microvascular endothelial cells in vivo, even though various IAV strains have been found to reproduce in human pulmonary microvascular endothelial cells.^218^, ^219^ Importantly, one research discovered that stopping the highly pathogenic H5N1 IAV strain from replicating in the endothelium lowered systemic viral dissemination and death without altering viral replication in infected mice's lungs.^220^ In a recent investigation, human capillary organoids generated from induced pluripotent stem cells were shown to be infected with SARS‐CoV‐2, which was prevented by using soluble, recombinant human ACE2.^221^ When compared to noninfected controls, COVID‐19 individuals who died exhibited higher expression of ACE2 in pulmonary endothelial cells.^222^ SARS‐CoV‐2 infection of the endothelium was identified in two investigations, as well as diffuse endothelial inflammation in the lungs, heart, kidneys, and liver.^216^, ^222^ Endothelial cells infected with SARS‐CoV‐2 may undergo apoptosis or pyroptosis. The endothelium's putative function in COVID‐19 has been explored in recent studies.^223^, ^224^

#### 15.3.2 Neutrophils and NETs

Middle East respiratory syndrome coronavirus (MERS‐CoV) and SARS‐CoV individuals have hematopoietic alterations.^225^ SARS‐CoV individuals are neutrophilic, and leukocytosis is linked to a poor prognosis.^226^, ^227^ Individuals infected with MERS‐CoV have been shown to exhibit neutrophilia in other investigations.^228^, ^229^, ^230^ COVID‐19 individuals have a higher circulating neutrophil quantity, and a high neutrophil count has been linked to a bad prognosis.^231^, ^232^, ^233^, ^234^, ^235^

By phagocytosing viral particles and releasing NETs, neutrophils help to remove viruses from the lungs.^236^, ^237^, ^238^ Activated neutrophils, on the other hand, may cause harm to host cells.^239^, ^240^, ^241^, ^242^ Neutrophils are also important in immunothrombosis, which is the stimulation of coagulation that occurs as a result of the host's innate immunological response.^243^ NETs have been linked to thrombosis and vascular blockage.^244^, ^245^ Citrullinated histone H3 and myeloperoxidase (MPO)‐DNA complexes are two biomarkers applied to evaluate the number of NETs in plasma. Many of these tests, however, have limited specificity for NETs.^245^ MPO‐DNA complexes were shown to be higher after hospital admission in IAV H7N9 and H1N1 individuals in one investigation, which was linked to illness severity.^246^ Serum samples from COVID‐19 individuals with severe disease had higher amounts of citrullinated histone H3 and MPO‐DNA complexes.^247^ These findings show that NETs may play a role in the blood flow problems of the lungs of individuals with COVID‐19.^245^, ^246^, ^247^, ^248^

#### 15.3.3 Complement

By opsonizing viral particles, recruiting inflammatory cells, and killing infected cells, the complement system has a critical part in the host's immunological response to viruses.^249^

Complement activation, on the other hand, may harm the host's cells. The complement system is activated in mice infected with SARS‐CoV.^250^ There are strong hypotheses of the association between coagulation pathways and complement systems. Thus, inhibition of the complement system can be used as a potential target in the treatment of COVID‐19.^214^ It has been assumed that the binding of SARS‐Cov2 to mannose‐binding lectin‐associated serine protease (MASP2) can triggers complement activation and amplify tissue injury. Activating the complement pathway brings about increased endothelial cytokines production including MCP‐1, IL‐8, and IL‐1 along with RANTES, and additionally upregulates the endothelial essential adhesion molecules expression such as vWF and P‐selectin.^251^, ^252^ Moreover, CRP is described as an inflammatory marker that activates the complement pathway.^253^ In comparison to controls, C3/mice had decreased neutrophil and inflammatory monocyte migration into the lungs and less respiratory disorder following SARS‐CoV infection.^250^ In hDPP4 mice infected with MERS‐CoV, inhibiting the C5a receptor decreased lung damage.^254^ These findings suggest that the complement system had a role in lung disease in mice infected with MERS‐CoV and SARS‐CoV. Importantly, COVID‐19 individuals’ lung microvasculature showed substantial terminal complement component deposition.^255^

#### 15.3.4 von willebrand factor activation

Sticking subendothelial collagens and platelets together and activation of thrombosis and platelets aggregation on endothelial damage is the function of active von Willebrand factor which functions as a molecular glue.^214^ Despite some obstructions, it is reported that the elevation of the von Willebrand factor happens not only in critically ill patients but in non‐critically ill COVID‐19 patients as well, and in consequence, there is a possibility of having high thromboembolic risks for both non‐critically along with critically ill COVID‐19 patients.^256^, ^257^

## 16 COAGULOPATHY AND INFLAMMATION

The inflammatory response in COVID‐19 individuals is particularly striking, with prolonged fever, increased inflammatory markers levels (such as ferritin, ESR, various cytokines, and CRP, including TNF, IL‐1, as well as IL‐6), and a hyperinflammatory immune response called a cytokine storm, which is linked to poor outcomes.^232^, ^258^, ^259^, ^260^, ^261^ According to early findings, this inflammatory response was once thought to be similar to that found in haemophagocytic lymphohistiocytosis or macrophage activation syndrome.^247^, ^261^, ^262^, ^263^, ^264^ Later research has shown distinct profiles of macrophage, neutrophil, lymphocyte, and other immunological responses in COVID‐19 individuals, as well as indications of complement activation and NeTosis.^247^, ^261^, ^262^, ^263^, ^264^ The link between higher inflammatory cytokine circulating levels and abnormal coagulation measures is an interesting observation.^258^, ^265^ In COVID‐19 individuals, plasma IL‐6 levels have been demonstrated to relate directly to fibrinogen levels.^266^ The pathophysiology of COVID‐19 is considered to be influenced by a severe inflammatory state and thromboinflammation with hypercoagulability.^265^, ^266^, ^267^ When compared to healthy people, COVID‐19 individuals had higher levels of prothrombotic acute phase reactants including von Willebrand factor, fibrinogen, along with factor VIII, which are often raised during inflammatory situations. COVID‐19 implicates the endothelium, platelets, and coagulation system as possible coagulopathy and thrombosis mediators.^198^, ^268^, ^269^, ^270^ As a result, combining innovative anti‐inflammatory techniques with antithrombotic medications, such as IL‐1 or IL‐6 signaling antagonists, may have a larger benefit in avoiding thrombosis and mortality in COVID‐19 individuals than either therapy alone.^271^, ^272^, ^273^

Severe COVID‐19's common feature which is increasingly evident is its hypercoagulable disorder.^274^ Although COVID‐19 can affect all coagulation parameters, the amount (level, degree) of these changes and their connection with disease mortality and severity is considerably variable.^231^, ^274^, ^275^ In hospitalized patients, a coagulation profile should be prepared for confirmed or suspected cases, including platelet count, partial thromboplastin time (PTT), prothrombin time (PT), platelet count, D‐dimer, and fibrinogen.^276^ VTE has been found to occur in 10%–35% of COVID‐19 individuals, with an autopsy showing that it might reach over 60%.^277^, ^278^, ^279^, ^280^, ^281^ Despite earlier indications that individuals with COVID‐19 had a greater prevalence of thrombosis than individuals with other forms of pneumonia, subsequent research indicated that the VTE incidence in individuals with COVID‐19 was 2%, compared to 3.6% in individuals with non–COVID‐19 community‐associated pneumonia.^282^ The pathological results from autopsies of individuals infected with the pandemic of coronaviruses have been described in many publications. SARS‐CoV individuals had pulmonary thrombi, thrombi in tiny arteries, and fibrin inside pulmonary vessels.^283^, ^284^, ^285^ Fibrin thrombi were seen in the lungs’ small arteries and capillaries, as well as hemorrhage foci.^255^, ^286^ Within alveolar capillaries, CD61^+^ megakaryocytes were discovered.^286^ Neutrophils were also linked to certain fibrin and platelets found in tiny arteries. A subgroup of individuals with severe COVID‐19 had intra‐alveolar fibrin depositions, indicating a reduction in vascular integrity.^287^ According to one autopsy investigation, 7 of 12 COVID‐19 individuals (58%) had a DVT that was not identified as antemortem, also PE was the primary cause of death in four of these individuals.^281^ Autopsies were obtained on seven individuals with COVID‐19, and the results were compared to seven individuals with H1N1 in recent research. In the lungs of individuals with COVID‐19, there was extensive thrombosis and microangiopathy, and microthrombi in capillaries were nine times more common than in H1N1, suggesting a distinct pathogenic mechanism.^222^

VTE was found in 15% of individuals with COVID‐19 in a recent extensive meta‐analysis of 44 trials focusing on severe complications and death in 14,866 hospitalized individuals with COVID‐19; however, the authors acknowledge extremely low‐quality data owing to substantial heterogeneity and bias risk.^288^ Utilizing a random‐effects method, a recent systematic study of 28 papers reporting 397 DVT occurrences in a total of 4138 individuals with COVID‐19 revealed that the pooled analysis of the incidence for DVT was 16%.^289^ Individuals with COVID‐19 from China (30%) had a substantially higher pooled incidence of DVT (*p* < 0.01) than those from Western nations (13%) based on their geographic location. In individuals with COVID‐19 admitted to the ICU, the pooled incidence of DVT was substantially greater (23%) than in individuals with COVID‐19 who did not need ICU monitoring (5%, *p* < 0.01). The cumulative prevalence of the composite result (DVT, PE, MI, ischemic stroke, or systemic embolism of arterial) was 31% in a recent investigation of 184 ICU individuals with COVID‐19‐related pneumonia, of whom 23 cases died (13%), 22 cases were discharged alive (12%), and 139 cases (76%) were still in the ICU, all receiving thromboprophylaxis.^290^ Moreover, DVT screening was performed on days 5 through 10 of admission and revealed a 32% frequency of VTE among 25 severely sick individuals with COVID‐19 admitted to the ICU; proximal DVT was found in 6 cases (24%) and PE in five cases (20%).^291^

According to a meta‐analysis of 11 cohort studies, 23.9% of COVID‐19 individuals who were hospitalized had VTE despite anticoagulation. In addition, PE was discovered in 11.6% of individuals, while DVT was diagnosed in 11.9%. VTE was found to be more common among ICU individuals (30.4%) than on the ward (13%).^292^ Moreover, a meta‐analysis of 12 investigations found that the pooled VTE incidence among ICU individuals was 31% (95% confidence interval [CI] 20%–43%), with all individuals receiving low‐molecular‐weight or unfractionated heparin for thromboprophylaxis.^293^

Based on a retrospective cohort analysis of 289 individuals (average age: 62.2 years, 41% female) admitted to general hospital wards with positive COVID‐19, VTE imaging examinations were done in 100 individuals (34.6%), and VTE was found in 49 individuals (17%). In addition, PE was found in 42 individuals (14.5%), cerebral sinus venous thrombosis in three individuals (1%), and DVT in twelve individuals (4.2%). A total of 90 individuals (31%) died or were sent to the ICU, which was nearly 2‐fold greater among VTE individuals (47.9% vs. 27.9%, *p* = 0.01). In non‐ICU individuals, a thromboprophylaxis deficiency was a key factor of VTE in individuals with COVID‐19.^294^

## 17 THROMBOSIS TARGETING FOR THE TREATMENT OF COVID‐19

The high thrombotic complications incidence seen in critically ill patients of COVID‐19 has generated considerable interest in the application of antithrombotic drugs for COVID‐19 patients.^295^

## 18 ANTICOAGULATION

The significant increase in the prevalence of thrombotic complications has led many hospitals to routinely administrate strict VTE prophylaxis by using either unfractionated heparin (UFH) or LMWH, and in several groups, the full or intermediate dose of anticoagulant was used to prevent these thrombotic complications.^296^, ^297^

Anticoagulants prevent coagulation via antithrombin‐mediated inhibition of FXa or thrombin.^295^ Nevertheless, in addition to its anticoagulant effects, heparin seems to have pleiotropic effects that might offer unique benefits in infection, such as anti‐inflammatory effects through binding to damage‐associated molecular patterns (DAMPs) such as high mobility group box 1 (HMGB1) and pro‐inflammatory cytokines.^298^, ^299^ Recent research directly investigated the relationship between heparin and SARS‐CoV2 and showed that heparin might have antiviral properties because it can directly bind to the SARS‐CoV2 virus spike protein.^300^

## 19 INHIBITION OF THE CONTACT FACTOR ACTIVATION PATHWAY

FXII Inhibition as a means of suppressing the thromboinflammatory response due to severe COVID‐19 seems to be an attractive and reasonable therapeutic target.^295^ Treatment via an inhibitory antibody of FXIIa leads to inflammatory markers decrement such as neutrophil degranulation, IL‐6, and complement activation.^301^, ^302^, ^303^ An increment of negatively charged molecules like NETs in severe COVID‐19 cases can activate FXII which initiates Contact pathway activation.^304^ This results in the formation of thrombin and bradykinin activation, in particular, the latter leads to the subsequent activation of complement and the production of the inflammatory cytokines.^305^

## 20 ANTIPLATELET AGENTS AS POTENTIAL THERAPEUTICS FOR COVID‐19

A recent meta‐analysis of randomized controlled trials found no benefit in adding antiplatelets to COVID‐19 treatment.^306^ Strangely, most of the data linking clinical outcome improvement with antiplatelet therapy relate to aspirin.^307^ In this respect, experimental data show that targeting platelets via aspirin precludes neutrophil‐mediated microvascular thrombosis and intravascular coagulation in a murine model of bacterial sepsis.^308^ Dipyridamole and several other antiplatelet drugs, including nafamostat, are currently being appraised for their potential part in decreasing the COVID‐19 severity.^295^

## 21 CONCLUSION

VTE is a common complication of viral diseases. Since the rise of COVID‐19 cases, clinicians paid more attention to the diagnosis and treatment of VTE associated with viral diseases, especially COVID‐19. Coagulation disorders such as diffuse intravascular coagulation, pulmonary thromboembolism, and DVT are reported in viral infections. Notwithstanding the unclear pathophysiology of viral infections, neonates with poor prognosis compared to adults and due to disagreement about the prescription and usefulness of antiviral and anticoagulant therapy in patients, it seems that viral infections may cause significant disruption of intrinsic coagulation and anticoagulation processes in patients. Endothelial dysfunction, excessive inhibition of fibrinolysis, increased blood viscosity, sepsis‐associated coagulopathy on the arterial, venous, and microcirculatory systems, platelet dysfunction, and higher levels of D‐dimer, as well as inflammatory activation, should be considered in viral infections. Clinicians should be in charge of developing an appropriate treatment plan with the patient's clinical condition to control and prevent possible complications and the occurrence of thromboembolism in viral diseases. Table 2 summarizes all the changes occurring by viral infections for each virus.

**Table 2:** Mechanisms involved in the association between viral diseases and VTE.

Virus	Mechanisms of causing VTE
Human Immunodeficiency Virus	Low CD4 cell count by inducing hypercoagulable condition^41^ Immune reconstitution inflammatory syndrome upon starting antiretrovirals can lead to hypercoagulable condition and increased risk of VTE^55^, ^56^, ^57^ Concomitant infection with mycobacterium Avium, mycobacterium tuberculosis, CMV, and pneumocystis Jiroveci pneumonia induce a hypercoagulable state which leads to VTE. In addition, mycobacterium tuberculosis promotes the activation of cytokines such as IL‐1, IL‐6, and TNF‐α.^62^ Higher viral load (HIV RNA) increases the risk of VTE^51^ HIV patients are at higher risk of Kaposi Sarcoma which increases the risk of VTE^74^ Age is an independent risk factor for VTE and in HIV patients, the median age of VTE is lower than the general population^76^ Activation of D‐dimer, CRP, soluble TNF receptors, and IL‐6 produce hypercoagulable conditions^80^ Higher anticardiolipin antibodies in HIV patients lead to a higher incidence of VTE^82^, ^83^ HIV damages endothelium causing increased activation of plasminogen activator inhibitor‐1, vWF, tissue factor, P‐selectin, and factor V which increases VTE incidence^92^, ^93^ P‐selectin is correlated with VTE in HIV patients^104^ A higher number of monocytes expressing tissue factor was detected in HIV patients^60^ HIV patients with VTE had higher protein C deficiency^108^ HIV patients with VTE had antithrombin deficiency which may be associated with a higher incidence of VTE^110^
Cytomegalovirus	CMV upregulates the expression of VCAM‐1, ICAM‐1, and tissue factor which leads to higher VTE by platelet aggregation, thrombin formation, and factor X activation^152^, ^153^, ^154^, ^155^ CMV increases VTE incidence by increasing vWF and factor VIII^157^, ^158^ Transition hypercoagulable state occurs by the production of APS and anticardiolipin antibodies
Zika and Chikungunya	Higher levels of D‐dimer were detected in Zika (19.4%) and Chikungunya (63.8%) patients which may cause higher VTE in these patients^6^
Epstein‐Barr	Factors associated with VTE were normal in EBV^187^
Varicella‐Zoster	Lower qualitative and quantitative antithrombotic proteins was responsible for primary thrombophilic situation in VZV patients with VTE^14^
Hepatitis C	Vasculopathy caused by chronic inflammation^11^, ^12^ Presence of anticardiolipin and antiphospholipid antibodies^11^, ^12^ Downregulation of anticoagulant proteins and upregulation of procoagulant proteins^11^, ^12^ Higher thrombin generation rates^11^, ^12^ Higher cryoglobulinemia which can cause thrombotic vasculitis^11^, ^12^
Herpes Simplex	Reduction in synthesis of heparan sulfate proteoglycan, increase in thrombin generation and platelet binding, decrease in prostacyclin synthesis and preventing binding of antithrombin to its surface^9^
COVID‐19	Elevated fibrin deposition inside the vascular and alveolar space caused by increased levels of plasminogen activator inhibitor 1^202^, ^203^ SARS‐CoV‐2 reduce ACE2 activity which decreases angiotensin I and XII (anti‐thrombotic and anti‐inflammatory) while increases angiotensin II (prothrombic and pro‐inflammatory)^214^ SARS‐CoV‐2 induces endothelium dysfunction which may contribute to occurrence of VTE^223^ Higher levels of neutrophils and NETs contribute to higher VTE^226^, ^227^ SARS‐CoV‐2 activates complement system which elevates rate of VTE^250^ Elevated vWF results in higher thromboembolic risk^256^, ^257^ Higher inflammatory cytokine levels (IL‐1, IL‐6, vWF, fibrinogen, and factor VIII) are associated with abnormal coagulation^258^, ^265^

Abbreviations: ACE2, angiotensin‐converting enzyme 2; APS, antiphospholipid syndrome; CMV, cytomegalovirus; COVID‐19, coronavirus disease of 2019; CRP, C‐reactive protein; EBV, Epstein‐Barr virus; HCV, hepatitis C virus; HIV, human immunodeficiency virus; HSV, Herpes simplex virus; ICAM, intracellular adhesion molecule; IL, interleukin; NET, neutrophil extracellular trap; SARS‐CoV‐2, severe acute respiratory syndrome coronavirus 2; TNF, tumor necrosis factor; VCAM, vascular cell adhesion molecule; VTE, venous thromboembolism; vWF, von Willebrand factor; VZV, Varicella‐Zoster virus.

## AUTHOR CONTRIBUTIONS

**Nasibeh Zerangian**: Investigation; methodology; writing – review and editing. **Gisou Erabi**: Methodology; writing – original draft; writing – review and editing. **Mohadeseh Poudineh**: Conceptualization; writing – original draft. **Kosar Monajjem**: Investigation; writing – original draft. **Maryam Dianati**: Conceptualization; writing – review and editing. **Maryam Khanlari**: Investigation; resources. **Diba Allafi**: Methodology; writing – original draft. **Arezoo Faridzadeh**: Project administration; validation; visualization. **Arian Amali**: Investigation; visualization; writing – original draft. **Nilufar Alizadeh**: Conceptualization; supervision. **Yasaman Salimi**: Project administration; resources. **Sajjad G. Ezabadi**: Investigation; writing – original draft. **Amir Abdi**: Methodology; writing – review and editing. **Niloofar Deravi**: Conceptualization; project administration; resources; supervision; validation.

## CONFLICT OF INTEREST STATEMENT

The authors declare no conflict of interest.

## TRANSPARENCY STATEMENT

The lead author Nilufar Alizadeh, Niloofar Deravi affirms that this manuscript is an honest, accurate, and transparent account of the study being reported; that no important aspects of the study have been omitted; and that any discrepancies from the study as planned (and, if relevant, registered) have been explained.

## Data Availability

Data are available upon request from the corresponding author.
